# Pelvic Hæmatocele; with a Somewhat Unusual Case

**Published:** 1908-08-15

**Authors:** 


### Gynæcology.

### PELVIC HÆMATOCELE: WITH A SOMEWHAT UNUSUAL CASE.

Pelvic Hæmatocele is so frequently the result of the rupture of a tubal gestation, or of the extrusion of a tubal mole through the abdominal ostium (tubal abortion), that other causes may be neglected. Whilst active and progressive bleeding gives rise to important symptoms, such as we associate always with rupture of an ectopic gestation, it must not be overlooked that a very considerable bleeding may occur without any sudden or even serious symptoms at the time. The explanation of such cases must be that the bleeding takes place slowly and is of the nature of general oozing or venous hæmorrhage rather than arterial. This kind of bleeding is more likely to occur in a tubal abortion than in a rupture of the tube; but this cannot be taken as an invariable rule, because sometimes quite small hæmatoceles form just at the site of a rupture.

The true nature of these cases may be overlooked, and they may be mistaken for ordinary uterine abortions. This mistake arises because uterine bleeding almost always occurs soon after the conversion of the tubal pregnancy into a mole and its subsequent extrusion. Such metrorrhagia in one who has perhaps missed a menstrual period very naturally suggests a miscarriage, and unless very careful examinations are made the true nature of the case may be overlooked.

The uterine bleeding in these cases may persist for many days or even weeks. The reason for this is that there is marked uterine congestion, and the decidua which has been formed in the uterus may not be completely thrown off. A case recently under the care of the writer is a good example of this, and presents some other points of interest in addition. The patient, a multipara, missed a period and later commenced to bleed freely from the uterus. She also suffered severe pain, thought to be due to uterine contractions, and not at any time so severe as to cause fainting or collapse. At first there was no palpable pelvic swelling, but towards the end of a week an indefinite mass was felt which was mistaken for an enlarged uterus. Bleeding continued for a fortnight, accompanied by very severe pain, the patient becoming very seriously ill and having rises of temperature to about 102° Fahr.

When seen at the end of the fortnight in consultation, an ill-defined mass was found reaching to the level of the umbilicus. This mass was more marked on the right side than on the left. It was sub-resonant all over upon light percussion, but dulness could be obtained on deeper pressure. It was clear, therefore, that whatever the mass was it had intestine adherent over it.

Per vaginam the uterus was found to be pushed up out of the pelvis towards the symphysis pubis, the cervix being very high up, and the fundus not to be differentiated from the mass, by which it was enveloped. Behind the cervix a soft boggy mass could be felt which the finger indented to some extent, and per rectum this mass seemed to bulge the anterior rectal wall. The whole mass was very tender. The patient was anæmic, with dry lips and sordes upon the teeth; she was extremely ill with a quick pulse and a temperature of 102°. A provisional diagnosis of pelvic hæmatocele was thereupon made, and she was operated on as soon as possible.

On opening the abdomen the mass was found to consist of blood-clot and serum, with a false capsule of inflammatory tissue to which coils of intestine were adherent. After carefully packing off the general peritoneal cavity with gauze wherever it could be seen or felt, some of the adhesions were separated and purulent fluid began at once to flow out; this condition had not been suspected, although the fever and general condition of the patient should have led to the diagnosis. A large opening was then made into the sac and two handfuls of old stinking blood-clot were removed, along with about a pint or a pint and a half of foul smelling fluid. The sac was wiped dry, and as the patient was in no condition to stand a prolonged operation, an opening was at once made into the vagina through the posterior fornix, and a large drainage tube put through and brought out at the abdominal wound. Gauze packing was applied around this so as to close the general peritoneal cavity, and the abdominal wound was then sutured around the tube and packing. The patient was placed upright in bed in the Fowler position, and saline rectal injections were given every four hours. Her condition at once began to improve, and after the operation she had no bad symptom of any kind.

The drainage tube was drawn down into the sac after forty-eight hours, and was pulled lower each day into the vagina; it was finally removed at the end of six days. The track was irrigated daily with hydrogen peroxide, and the foul discharge quickly ceased. The vaginal sinus had completely closed at the end of three weeks, when the patient got up, and the abdominal wound was also soundly healed. Three months later the patient was in perfect health and had no pain or discomfort whatever in the pelvis.

It is interesting to note that the gravid fallopian tube was not found at the operation; no attempt was made to find it, for fear of disseminating the infection. In spite of this, however, the treatment adopted cured the patient, and the same thing is seen in hæmatoceles incised and drained per vaginam. The infection in this case was caused by the bacillus coli communis, which was cultured from the fluid in the sac. Presumably the organisms found entrance through the wall of the rectum or sigmoid. Suppurating hæmatoceles are rare conditions and decomposing contents occur very seldom.

